# AAV Induced Expression of Human Rod and Cone Opsin in Bipolar Cells of a Mouse Model of Retinal Degeneration

**DOI:** 10.1155/2021/4014797

**Published:** 2021-02-09

**Authors:** Michelle E. McClements, Federica Staurenghi, Meike Visel, John G. Flannery, Robert E. MacLaren, Jasmina Cehajic-Kapetanovic

**Affiliations:** 1 Nuffield Laboratory of Ophthalmology, Department of Clinical Neurosciences & NIHR Oxford Biomedical Research Centre, University of Oxford, UK; 2 Helen Wills Neuroscience Institute, University of California, Berkeley, CA, USA; 3 Oxford Eye Hospital, Oxford University Hospitals NHS Foundation Trust, Oxford, UK

## Abstract

Vision loss caused by inherited retinal degeneration affects millions of people worldwide, and clinical trials involving gene supplementation strategies are ongoing for select forms of the disease. When early therapeutic intervention is not possible and patients suffer complete loss of their photoreceptor cells, there is an opportunity for vision restoration techniques, including optogenetic therapy. This therapy provides expression of light-sensitive molecules to surviving cell types of the retina, enabling light perception through residual neuronal pathways. To this end, the bipolar cells make an obvious optogenetic target to enable upstream processing of visual signal in the retina. However, while AAV transduction of the bipolar cells has been described, the expression of human opsins in these cell types within a model of retinal degeneration (*rd1*) has been less successful. In this study, we have expanded the optogenetic toolkit and shown successful expression of human rhodopsin driven by an ON-bipolar cell promoter (*Grm6*) in the *rd1* mouse model using modified AAV capsids (AAV2.4YF, AAV8.BP2, and AAV2.7m8) delivered via intraocular injection. We also show the first presentation of ectopic expression of human cone opsin in the bipolar cells of *rd1* mice. These data provide evidence of an expansion of the optogenetic toolkit with the potential to restore useful visual function, setting the stage for future trials in human patients.

## 1 Introduction

Inherited retinal degenerations are the leading cause of blindness in the working age population, affecting approximately 1 in 4,000 people worldwide [1]. Numerous clinical trials have been initiated in recent years, providing adeno-associated viral (AAV) vector gene supplementation strategies for particular forms of inherited retinal disease [2–4], leading to the first FDA-approved gene therapy product, Luxturna. However, inherited retinal degeneration can result from different mutations in more than 250 genes (RetNet https://sph.uth.edu/retnet/), and many identified forms have very low prevalence. This makes it unfeasible to develop gene-specific treatments for all forms of inherited retinal degeneration. Additionally, the mode of inheritance of retinal degenerations is also varied and influences the opportunity for gene therapy intervention by current methods. However, the progression of disease exhibits a similar form in many patients regardless of the genetic origin or pattern of inheritance, typically consisting of loss of rod photoreceptor cells followed by loss of cone photoreceptor cells, amounting to a loss of the outer layer of the retina. Despite the loss of the light-sensitive photoreceptor cells, inner retinal cells survive and remain largely functional, making them a potential target for optogenetic strategies, a universal approach that can be applied regardless of the genetic origin of the disease [5].

Bipolar cells transfer the light signals received from photoreceptor cells to amacrine and retinal ganglion cells. Selective expression of optogenes in ON-bipolar cells, as opposed to nonspecific pan-neuronal expression with ubiquitous promoters, is considered important to avoid interfering with the complex interconnected signalling pathways of the neural retina. However, efficient transduction of the inner retinal layers, and in particular the ON-bipolar cells, has proved difficult to date as the majority of gene therapy treatments have been designed to target the photoreceptor cells and/or retinal pigment epithelium via subretinal delivery route. Moreover, intravitreally delivered wild-type AAV vectors have encountered difficulties in penetrating the retina due to naturally existing ocular barriers, including the inner limiting membrane found at the critical vitreo-retinal interface [6, 7]. This membrane is several folds thicker in primates—an important consideration for translational studies. To this end, the use of adjunctive glycosidic enzymes to tackle the retinal barriers to AAV transduction has significantly increased the expression profile of AAV vector-mediated retinal transduction in *rd1* mice including some expression in bipolar cells [7, 8]. In addition, increased tropism and transduction success in the retina have been improved by use of alternative AAV vectors. Thus, primate-derived recombinant AAV [9], AAV capsid mutants such as AAV2/2 (4YF), which avoid proteosome degradation [10], or directly evolved (AAV2/2 (7 m8) and AAV2/8 (BP2)) variants, which enhance retinal penetration, have enabled transduction of ON-bipolar cells in *rd1* mice [11, 12]. This targeting has been aided by optimisation of the *Grm6* ON-bipolar cell promoter, a gene that encodes the metabotropic glutamate receptor type 6 (mGluR6), associated with ON-bipolar cell activity [13, 14].

Various opsins have been used for optogenetic approaches [15] with a focus on microbial opsins [12, 16–18]. However, the potential of adverse immune responses elicited by microbial opsins raise doubts about their safety profile when used in humans so use of humanised versions [12, 17, 18] (NCT02556736, NCT03326336) or a human protein [19–23] may be more desirable. In addition, human opsins work at physiological light levels through G-protein coupled cascades, which lead to signal amplification, adaptation, and enhanced sensitivity. Despite encouraging data achieved with intravitreal rhodopsin optogenetic therapy in restoring vision in blind *rd1* mice [21, 22], the human rhodopsin expression in the inner retina remains somewhat limited and patchy, even when AAV vector is delivered in conjunction with glycosidic enzymes that enhance its retinal transduction [21] or when using the AAV2/2 (4YF) enhanced vector [22]. Optogenetics is a rapidly advancing field and here is aimed at expanding the toolkit available for such approaches, offering greater selective transduction efficiency of desired cell types with human opsin variants.

Herein, we provide proof-of-concept data that show robust human opsin-YFP expression can be achieved in bipolar cells in an advanced mouse model of retinal degeneration. We have confirmed the potential of the AAV2/2 (4YF) mutant capsid as well as identifying two further advanced capsid variants as candidates for bipolar cell transduction: AAV2/2 (7m8) and AAV2/8 (BP2). Our data also confirm these vectors enable bipolar cell expression of opsin fusion proteins when provided via intravitreal or subretinal delivery methods. All transgenes used the promoter, *4xGrm6-SV40,* previously shown to drive ON-bipolar cell specific expression of channelrhodopsins [11, 12]. In addition to confirming that human rhodopsin can be effectively targeted to bipolar cells using all three capsids and either intravitreal or subretinal delivery routes, we also provide the first presentation of medium-wave opsin (MWC) expression in bipolar cells of *rd1* mice. These data expand the options available for future optogenetic strategies and in particular encourage further investigations into the function and visual responses of human cone opsin in the degenerate retina.

## 2 Results

### 2.1 Expression of Human Rhodopsin Driven by an ON-Bipolar Cell Promoter Is Achieved by Both Intravitreal and Subretinal Injection of AAV2 (4YF)

Previous publications have described encouraging outcomes following intravitreal delivery to ON-bipolar cells in *rd1* mice [21, 22]. One study used wild-type AAV2 capsid and another the mutant AAV2 (4YF), both showing encouraging results. However, given the desire to translate preclinical studies to clinical trials, we hoped to identify a capsid that would enable expression of rhodopsin following subretinal delivery. As commonly used in other optogenetic strategies [22, 23], opsin-YFP fusion proteins were used throughout this study for direct visualisation of cells expressing the human opsin although opsin expression was also confirmed on selected eyes (Supplementary Figure 1). For comparison to the previously published studies, we tested the AAV2 (4YF) capsid and performed intravitreal injections as well as delivering our range of fusion vectors by subretinal injection.

**Figure 1:**
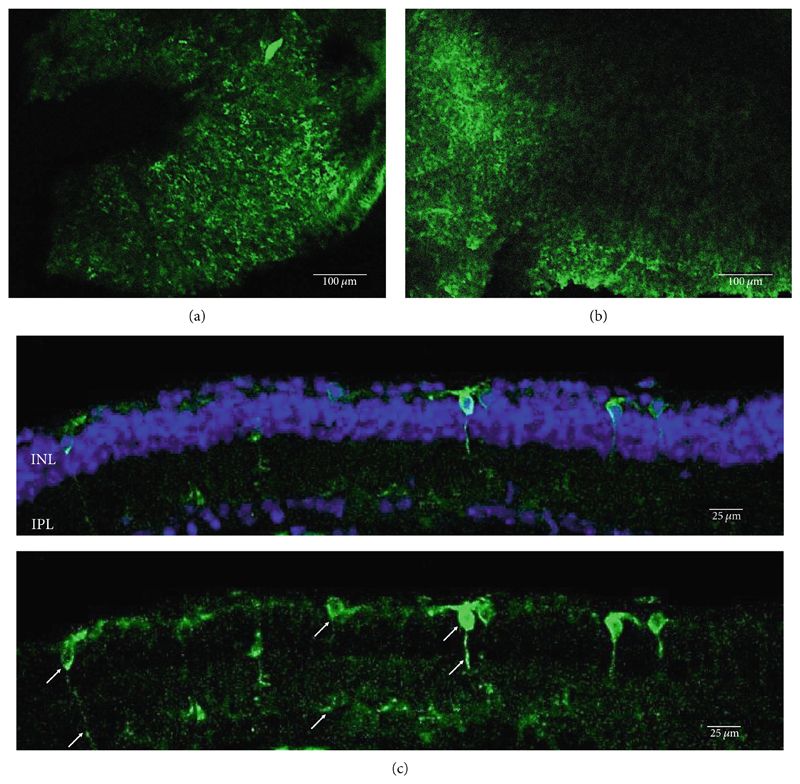
Rhodopsin expression in example *rd1* eyes following injection with AAV2/2 (4YF) 4xGrm6-SV40.RHO-http://YFP.WPRE.pA. Example tissue whole mounts revealed RHO-YFP expression in rd1 eyes after intravitreal injection (a) and subretinal injection (b) with retinal sections showing expression of the RHO-YFP fusion protein in retinal bipolar cells in the distal part of the inner nuclear layer (c). White arrows indicate select cells with bipolar cell morphology, including axon extensions towards the IPL. IPL: inner plexiform layer; INL: inner nuclear layer.

As has been previously reported, rhodopsin expression was successfully achieved from the ON-bipolar cell promoter following intravitreal injection with the AAV2/2 (4YF) vector, and we expand on this success by identifying the rhodopsin expression was achieved following subretinal delivery (Figure 1). Variability in expression was observed between the eyes but was apparent in most areas of the retina examined with the areas of highest expression shown. Retinal sections revealed the expression pattern of the fusion reporter was limited to the bipolar cells in the inner layer of the retina.

### 2.2 Expression of Human Rhodopsin Driven by an ON-Bipolar Cell Promoter Reveals Greater Transduction When Using AAV2 (7m8) and AAV2 (BP2) Capsids

**Figure 2:**
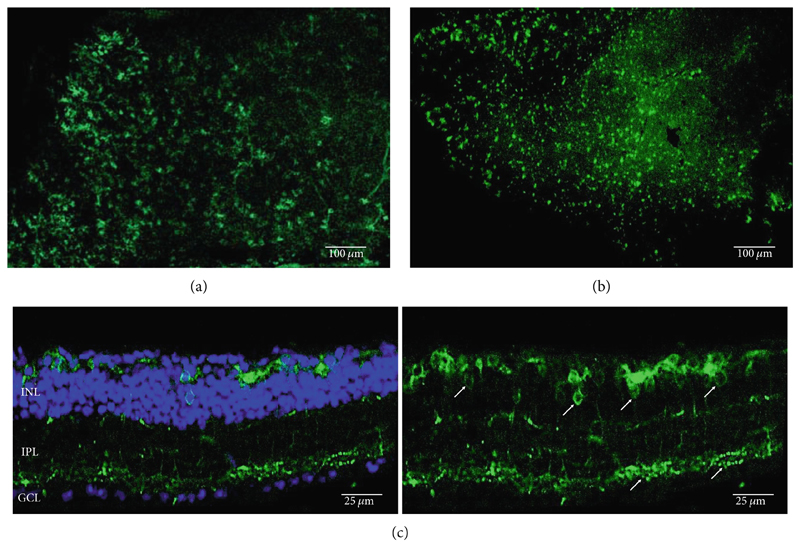
Rhodopsin expression in example *rd1* eyes following injection with AAV2/2 (7m8) 4xGrm6-SV40.RHO-http://YFP.WPRE.pA. Example tissue whole mounts revealed RHO-YFP expression in *rd1* eyes after intravitreal injection (a) and subretinal injection (b) with retinal sections showing expression of the RHO-YFP fusion protein in retinal bipolar cells in the distal part of the inner nuclear layer (c). White arrows indicate elected cells with bipolar cell morphology, including axon extensions towards the IPL with strong expression evident in the IPL. GCL: ganglion cell layer; IPL: inner plexiform layer; INL: inner nuclear layer.

**Figure 3:**
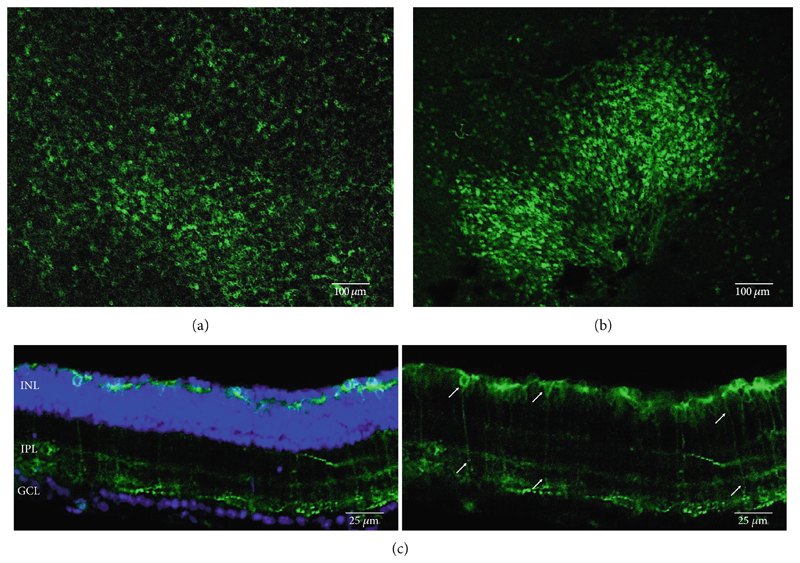
Rhodopsin expression in example *rd1* eyes following injection with AAV2/8 (BP2) 4xGrm6-SV40.RHO-http://YFP.WPRE.pA. Example tissue whole mounts revealed RHO-YFP expression in *rd1* eyes after intravitreal injection (a) and subretinal injection (b) with retinal sections showing expression of the RHO-YFP fusion protein in retinal bipolar cells in the distal part of the inner nuclear layer (c). White arrows indicate elected cells with bipolar cell morphology, including long axon extensions towards the IPL with strong expression also evident in the IPL. GCL: ganglion cell layer; IPL: inner plexiform layer; INL: inner nuclear layer.

Other studies attempting ON-bipolar cell expression with alternative optogenetic transgenes have used the novel capsids AAV2 (7m8) [12] and AAV2 (BP2) [11] or both [24]. However, no studies to date have published evidence of human rhodopsin expression in the retina using these capsid variants. In this study, we have observed expression using both intravitreal and subretinal injection methods with AAV2/2 (7m8) (Figure 2) and AAV2/8 (BP2) (Figure 3).

Both these capsid variants provided robust transduction success. As expected with use of the *4xGrm6-SV40* promoter, AAV2/2 (7m8) injected eyes consistently showed RHO-YFP expression in the bipolar cells within the inner retina (Figure 2). AAV2/8 (BP2) injected eyes similarly showed strong RHO-YFP fusion protein expression, which were more consistent through the injected area with long extensions towards the inner plexiform layer clearly evident (Figure 3).

### 2.3 Successful Expression of Human Medium-Wave Cone Opsin Driven by an ON-Bipolar Cell Promoter

**Figure 4:**
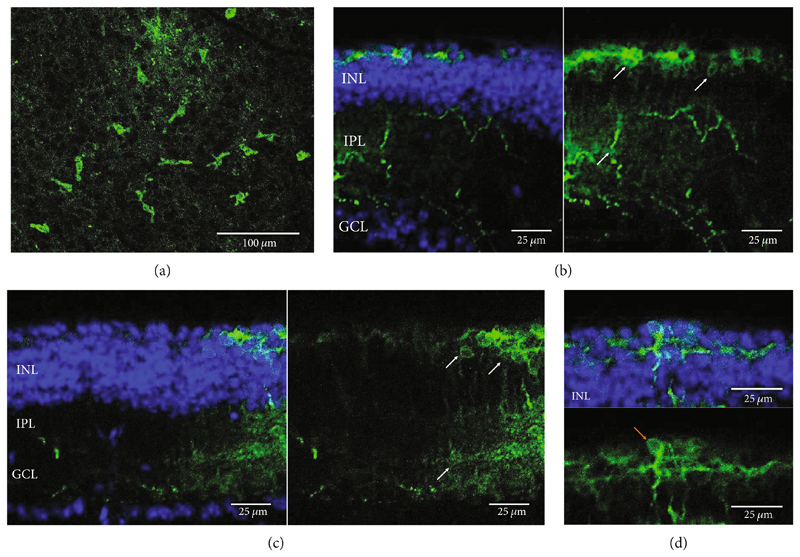
Medium-wave cone opsin expression in example *rd1* eyes following injection with AAV2/8 (BP2) 4xGrm6-SV40.MWC-http://YFP.WPRE.pA. Example tissue whole mounts revealed MWC-YFP fusion protein expression (a) with example retinal sections showing expression of the MWC-YFP fusion protein in retinal bipolar cells in the distal part of the inner nuclear layer (b, c). White arrows indicate elected cells with bipolar cell morphology, including axon extensions towards the IPL. Occasional suspected amacrine or horizontal cell expression was also observed (d), orange arrow. GCL: ganglion cell layer; IPL: inner plexiform layer; INL: inner nuclear layer.

The MWC opsin may offer some optogenetic advantages over human melanopsin and rhodopsin due to its quick recovery rate. However, expression of this opsin has only been achieved in retinal ganglion cells following intravitreal delivery of AAV2 (4YF) in *rd1* mice [23]. Achieving human cone opsin expression in the bipolar cells of the retina is an important step forward in expanding the optogenetic toolkit. Following our RHO-YFP expression comparisons with different capsid variants, we selected to use AAV2/8 (BP2) in our attempts to express a MWC-YFP fusion protein with the *4xGrm6* promoter. We show here for the first time, expression of human MWC opsin in the ON-bipolar cells of the retina of *rd1* mice (Figure 4).

## 3 Discussion

Our data provide evidence of the expanding toolkit for potential use in human optogenetic therapy with expression of human rhodopsin and MWC opsin achieved using an ON-bipolar cell promoter and advanced AAV capsid variants. We have used AAV2/2 (4YF), AAV2/2 (7m8), and AAV2/8 (BP2) to provide the first evidence of human rhodopsin expression with the ON-bipolar cell promoter *4xGrm6-SV40* following a clinically relevant subretinal injection delivery method (as used in current gene therapy clinical trials and without the use of adjunctive enzymatic treatments) in adult *rd1* mice. These mice suffer a fast onset of degeneration and at the time of injection would have no remaining rod photoreceptor cells and minimal cone photoreceptor cells [25]. The *4xGrm6-SV40* cell-specific promoter has previously been shown to be active in ON-bipolar cells in this mouse model [11, 12, 21, 22, 26], although one study using intravitreally delivered chimeric optogenetic protein struggled to confirm this in advanced and very late stage of retinal degeneration [24] suggesting potential degeneration-dependent downregulation of *Grm6* promoter driven gene expression. Additionally, expression from this promoter was also observed in other cell types including amacrine cells, an observation also made in this study with the apparent expression of MWC-YFP in such cell types (Figure 4). Despite this, the morphology of the majority of cells expressing the opsin fusion proteins used in this study predominantly resembled bipolar cells.

The implications of expressing human opsins in multiple cells are currently not well understood and require further investigations. The ideal scenario is to limit expression of such opsins to a single cell type to make best use of the interconnecting neural pathways of the surviving retina. It is possible that other ON-bipolar cell promoter options need to be considered in the future, and it has been shown that without the SV40 promoter, the mGluR6 enhancer with additional intron and promoter sequences enables enhanced reporter gene expression in ON-bipolar cells in mice and nonhuman primates following AAV delivery [27]. However, the resulting full promoter sequence is long, which may cause issues for future transgenes due to the restricted packaging capacity of AAV. In addition, a mini-promoter Ple155 (derived from the ON-bipolar *PCP2* gene) has also been shown to provide ON-bipolar cell expression [28, 29] but only in very young mice (at P2 and not at P30) with undifferentiated bipolar cells. It therefore remains to be seen whether the Ple155 promoter offers any advantage in ON-bipolar cell targeting over the *4xGrm6-SV40* in degenerate retina and whether *in vivo* ON-bipolar cell transduction can be achieved with either promoter in nonhuman primate or indeed in human retina.

Retinal remodelling following significant loss of photoreceptors including retraction of bipolar cell dendrites, synaptic rewiring, and changes in protein expression and trafficking [30, 31] will undoubtedly affect the success of any optogenetic therapy. Thus, careful selection of patients suitable for these therapies will be critical in order to achieve efficient bipolar cell targeting but before remodelling causes irreversible damage and scrambling of neural networks. However, the human inner retina appears to be relatively unaffected by the atrophy that disrupts the outer retina in advanced retinitis pigmentosa, and a proportion of patients show inner retinal thickening relative to control subjects [32]. While changes in some cell types reflect remodelling responses, many residual cells harbour molecular signatures that are typical of normal inner retinal cell morphology, including the bipolar cells, indicating a receptive environment for ectopic optogene expression with a capacity for plasticity [33].

In this pilot study, we highlight the expansion of optogene and capsid options available for optogenetic strategies targeting the distal retina following photoreceptor degeneration. For RHO-YFP and MWC-YFP fusion constructs, it is clear that successful expression using the *4xGrm6* promoter is achievable in the degenerate retina of the *rd1* mouse. The transduced cells showed long extensions through the inner nuclear layer and terminated at the inner plexiform layer, exhibiting clear ON-bipolar cell morphology [34]. It is known that rhodopsin expression in ON-bipolar cells can restore visual function in *rd1* mice [21] and that MWC opsin expression in retinal ganglion cells also provides functional rescue [23]. However, with improved transduction success by the combination of subretinal delivery and AAV2/2 (7m8) or AAV2/8 (BP2) vectors, the previous benefits achieved with rhodopsin may be improved while MWC opsin expression in the inner retina will be functionally explored in our future work. Importantly, subretinal optogenetic delivery will provide limited transduction of distal retinal circuitry which may produce a more natural image but is likely to require lower vector doses in human patients with lower risk of immunological stimuli and adverse intraocular inflammation [4].

In summary, we have confirmed the potential to express human opsins in retinal bipolar cells and provide the first presentation of human rhodopsin and medium-wave cone opsin expression following subretinal delivery with AAV2 (7m8) and AAV8 (BP2) capsids. These results pave the way for further experiments and investigations into the therapeutic potential of these optogenetic vectors.

## 4 Materials and Methods

### 4.1 Vector Design and Production

Human rhodopsin and medium-wave opsin sequences were fused to YFP to create transgenes of 4xGrm6-SV40.RHO-http://YFP.WPRE.pA and 4xGrm6-SV40.MWC-http://YFP.WPRE.pA. The transgenes were packaged in either AAV2 (4YF), AAV2 (7m8), or AAV8 (BP2) capsids using the standard triple transfection method and prepared in PBS [35]. Titres were achieved by qPCR targeting the ITRs common to all vectors.

### 4.2 In Vivo Injections

All animal procedures were conducted in accordance with the Association for Research in Vision and Ophthalmology (ARVO) statements on the care and used of animals in ophthalmic research. Adult male *rd1* mice (>6 weeks of age) were anaesthetised by intraperitoneal injection of ketamine (72 mg/kg) and xylazine (16 mg/kg). The pupils were fully dilated with 1% tropicamide and 2.5% phenylephrine eye drops, and a custom-made ultrafine needle (Hamilton RN needle, 34 gauge) was attached to a 5 *μ*l Hamilton glass syringe and passed at 45 degrees through the pars plana into the vitreous cavity (for intravitreal delivery) or into the subretinal space without retinal perforation (for subretinal delivery). Injections were performed under direct visualisation of the needle tip using an operating microscope (Leica Microsystems), avoiding lenticular contact and blood vessels. Each eye received a total intravitreal dose of 1E+14 genome copies in a 3 *μ*l volume or 2 *μ*l subretinal bleb (6 eyes per vector).

### 4.3 Tissue Preparations

At 6-8 weeks postinjection, the eyes were harvested and fixed in 4% paraformaldehyde for 1 hour at room temperature. Whole mounts were prepared by making a circular incision around the ora serrata, removing the cornea and gently manipulating the eye cup to remove the neural retina. For retinal cross-sections, whole mounts were embedded in agarose and sectioned transversely using a vibratome (Leica Microsystems) at medium speed, maximum vibration, and 100 *μ*m thickness. Whole mounts and sections were mounted on slides using VECTASHIELD antifade mounting medium (Vector Laboratories) containing DAPI to stain cell nuclei and examined under confocal microscopy to confirm expression of the opsin-YFP fusion proteins. For staining of rhodopsin, mounted sections were permeabilised with 0.2% Triton-X-100 PBS for 20 minutes at room temperature then blocked with 10% donkey serum (D9663, Sigma UK) in 0.2% Triton-X-100 PBS for one hour at room temperature. Primary antibody solution was then applied for 3 hours at room temperature (1 : 200 rabbit anti-human rhodopsin, Abcam ab112576 in blocking buffer). Sections were rinsed with 0.05% Tween20 PBS four times for 10 minutes each then incubated with secondary antibody solution for 2 hours at room temperature (1 : 200 AlexaFluor donkey anti-rabbit 488 in 2.5% donkey serum, 0.2% Triton-X-100 PBS). Slides were rinsed four times in 0.05% Tween20 PBS then once with water before being DAPI mounted as described above.

## Supplementary Material

Supplementary Figure

## Data Availability

The data used to support the findings of this study are included within the article.
