# Supplementary material for: AAV Induced Expression of Human Rod and Cone Opsin in Bipolar Cells of a Mouse Model of Retinal Degeneration
Source: Biomed Res Int. 2021 Feb 9;2021:4014797. doi: 10.1155/2021/4014797 (PMC7612646; doi:10.1155/2021/4014797)
Supplement: Supplementary file 1 — Supplementary Materials Supplementary Figure 1: rhodopsin staining in rd1 eyes injected with 4xGrm6-RHO with an absence of staining in a PBS injected eye. [file BMRI-2021-4014797-s001.docx]

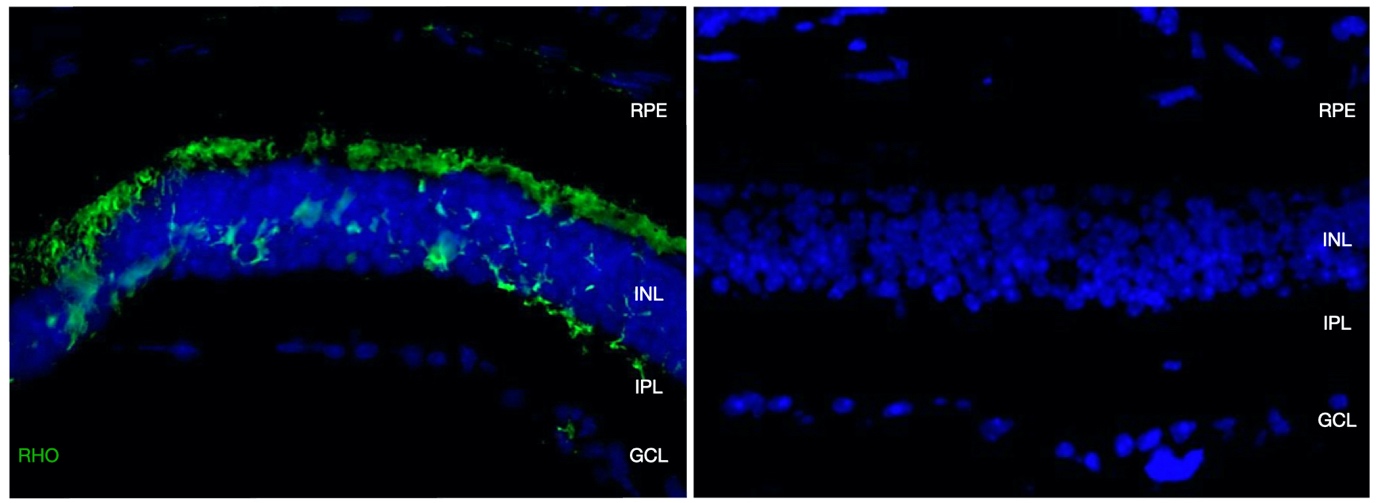


Supplementary Figure 1. Rhodopsin staining in *rd1* eyes injected with *4xGrm6-RHO* with an absence of staining in a PBS injected eye.
