# Supplementary material for: AAV Induced Expression of Human Rod and Cone Opsin in Bipolar Cells of a Mouse Model of Retinal Degeneration
Source: Biomed Res Int. Author manuscript; Available in PMC 2022 Apr 21. (PMC7612646; doi:10.1155/2021/4014797)
Supplement: Supplementary file 1 [file EMS144310-supplement-Supplementary_Figure.pdf]

Supplementary Materials

Supplementary Figure 1: rhodopsin staining in *rd1* eyes injected with *4xGrm6-RHO* with an absence of staining in a PBS injected eye. *(Supplementary Materials)*
